# Postoperative phlegmasia caerulea dolens: a case report and consideration of potential iatrogenic factors

**DOI:** 10.1186/1752-1947-1-163

**Published:** 2007-12-01

**Authors:** Ronan A Cahill, HP Redmond

**Affiliations:** 1 Department of Academic Surgery, NUI (Cork), Cork University Hospital, Wilton, Cork, Ireland

## Abstract

While the haemorrhagic consequences of anti-coagulants are well and frequently described in the surgical literature, the paradoxical prothrombotic tendencies of these drugs tend to be under-recognised due, perhaps, to their clinical infrequency. However, when these effects pertain, their consequences can be devastating. Here, we present a postoperative oncology patient who suffered a massive recrudescence of his lower limb venous thrombosis immediately after discontinuation of his heparin infusion, despite seemingly being adequately anticoagulated by warfarin therapy (INR > 2.0). We intend this case to graphically illustrate the theoretical considerations that must govern the perioperative use of these drugs in high-risk patients.

## Introduction

Despite increasingly effective regimens for its prophylaxis and prevention, venous thrombo-embolic disease remains a common and serious cause of both morbidity and mortality in surgical patients.[1] As predisposing and precipitating factors are often multiple in patients undergoing major surgery, a close understanding of all aspects of both this disease and its treatments is essential. It is particularly important to adhere to the principle of *primum non nocere *as certain preventative strategies may, paradoxically, induce a transient state of hypercoagulability.

Here, we present a patient with renal transitional cell carcinoma who suffered massive recrudescence of previous thromboembolic disease of his lower limb early after major surgery, despite receiving perioperative anticoagulants. The aim of this review, using this case as an illustration, is to highlight certain important considerations in the perioperative management of patients at high-risk of venous thrombosis.

## Case presentation

A 60 year old male presented with haematuria and anaemia, one month after commencing warfarin for treatment of a right lower deep venous thrombosis (DVT) with associated pulmonary embolus. Despite adequate oral anticoagulation (i.e. an INR > 2.0), he then developed a left lower limb DVT for which he was heparinised while his warfarin therapy was increased (INR > 2.5). Further investigations undertaken at this time to determine an underlying cause included an abdominal ultrasound and, subsequently, a computerized tomogram (CT), both of which revealed the presence of a solid mass, with appearances consistent with a renal carcinoma, arising out of his left kidney.

Given his propensity to intravascular thrombosis, it was decided to attempt operative resection of this cancer at the earliest opportunity recommended, and he was admitted for surgery one month after his last thrombotic episode. His warfarin was withheld, and he was commenced on a continuous infusion of heparin preoperatively. When his INR reached 1.5, he was scheduled for radical nephrectomy under general anaesthesia via a left subcostal incision. As is usual practice[2], his heparin was discontinued six hours before surgery and recommenced 12 hours after his operation. His oral anticoagulants were restarted on the first postoperative day. The histology of the resected specimens confirmed the presence of a locally advanced transitional cell carcinoma of his kidney with metastatic deposits in the regional lymph nodes.

**Figure 1:**
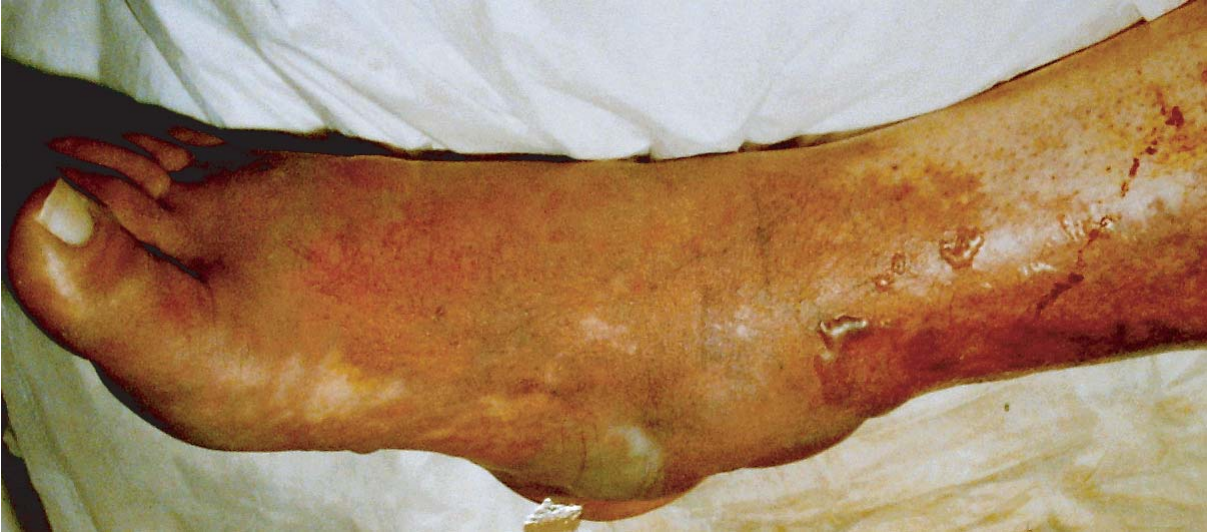
Photograph demonstrating the dorsum of the patient's right foot 24 hours after development of symptoms.

**Figure 2:**
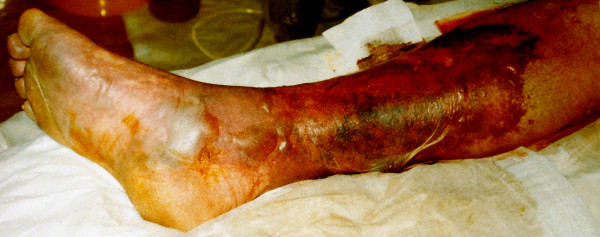
Photograph demonstrating the medial aspect of the patient's right foot and leg 24 hours after development of symptoms. Particularly evident in this photo is the marked discolouration of the skin as well as haemorrhagic blistering consistent with massive venous outlet obstruction.

On the third postoperative day, his INR exceeded 2.0 and his heparin was discontinued. However, that evening, he complained of an acute onset of severe right calf pain. This was associated with swelling, discolouration and coldness of leg that began distally and spread proximally although his peripheral pulses remained palpable (see Figures 1 and 2). A clinical diagnosis of *phlegmasia caerulea dolens *("blue, painful leg") with incipient venous gangrene was made and his heparin was immediately re-commenced and the affected limb elevated. Despite rapid fasciotomy, the condition of his limb deteriorated and he underwent a below knee amputation.

## Discussion

When multiple risk factors for venous thromboembolism co-incide, they exert a cumulative effect making the perioperative management of such patients crucially important. While the associated risk of pulmonary embolus is a commonly cited cause for concern, the venous occlusion itself can result in devastating consequences, both in the short-term (culminating, as in this patient, with the total occlusion of the venous drainage of the extremity)[3] and in later life (post-thrombotic limb syndrome).[4]

In addition to the several risk factors inherent in this man's disease process (immobility; malignancy; previous, recent venous thrombosis)[1,5], his treatment contributed others:

- General anaesthesia in itself is a risk factor for venous thrombus formation. In addition to reduced fibrinolysis[6], reduced arterial blood supply to the lower limbs may induce hypoxia with release of prothrombotic factors.[7]

- Intraoperatively, veins may be compressed either at the site of operation or distant from it (e.g. calf veins are compressed due to the loss of surrounding muscle tone). In addition, elimination of the "muscle pump" in the extremities leads to venous stasis.

- Heparin's main anticoagulant effects are mediated via the potentiation of antithrombin III.[8] However, after stopping heparin, levels of antithrombin III are reduced[9], which may be a factor in disease reactivation at this critical time[10], particularly if the thrombosis had only been incompletely suppressed. Furthermore, following abrupt discontinuation of heparin treatment, there can arise a transient increase in thrombin activity associated with a increase in activated protein C function.[11]

- Warfarin affects the synthesis of both procoagulant (factors II, VII, IX and X) and anticoagulant proteins (protein C and its co-factor protein S).[12] In the initial stages after commencement of warfarin, production of both factor VII along with that of proteins C and S are reduced. Given that this fall precedes that of Factor II, IX and X, the overall effect is to mimic that of familial thrombophilia and is prothrombotic. This process is thought to be clinically important in the pathogenesis (via microcirculatory occlusion) of warfarin-induced skin necrosis. The effect persists until the circulating levels of the other clotting factors diminish which may not be complete until four days after treatment begins[13] and it is only then that the drug's expected anticoagulating effects are invoked. In addition, following cessation of warfarin therapy, circulating levels of factors VII and IX recover faster than those of protein C and S, again inducing a potentially hypercoaguable state.

-The INR is most sensitive to levels of factor VII. The fact that full anticoagulation depends on depletion of all the vitamin K dependent clotting factors, means that this test is an inaccurate guide to the adequacy of the drug's effect during the first 36–48 hours of warfarin therapy.[13]

- Heparin therapy may cause prolongation of the INR[14], while warfarin may increase the APTT.[15] Therefore either of these tests may overestimate the effect of continuing therapy after cessation of the other drug.

The timing of our patient's complication suggests that both his ongoing thrombotic tendencies combined with an iatrogenically-induced decrease in antithrombotic activity to precipitate his massive venous occlusion. This phenomenon has been described previously in patients suffering acute coronary insufficiency, but, to our knowledge, has not been previously reported in patients with acute peripheral vascular thrombotic disease.

## Conclusion

Our case, therefore, emphasizes the importance of close attention to the correct perioperative handling of even seemingly familiar agents, particularly with regard to the use a sufficient period of overlap during which both agents are used in situations when anticoagulation is initiated by heparin and continued by warfarin. While the need to overlap treatments is often dryly advocated in guidelines, assumptions of the benign nature of the drugs early after their initiation (supported by the rarity with which problems are encountered empirically) combined with both hospital and patient-related factors to minimize hospital stay after diagnosis and initiation of treatment may erode adherence to theoretical concerns. We hope that this case serves to graphically reinforce the crucial pharmacological and physiological principles underlying the transition period between initial heparinisation and subsequent warfarin therapy.

## Competing interests

The author(s) declare that they have no competing interests.

## Authors' contributions

RC and HPB conceived of the report jointly-RA performed the initial drafting while HPB reviewed and perfected the manuscript. Both authors read and approved the final manuscript.

## Consent

Written informed consent was obtained from the patient for publication of this case report and any accompanying images. A copy of the written consent is available for review by the Editor-in-Chief of this journal.
